# Exploring the effects of holistic performance training in tactical athletes: a pilot longitudinal study of the game plan training programming

**DOI:** 10.3389/fspor.2026.1879693

**Published:** 2026-06-22

**Authors:** Jessi Truett, Leigh Ann Dodd, Kenneth Kersey, Mary Golden, Achraf Cohen, Mangesh Kulkarni, Adam Anz

**Affiliations:** 1 Behavior lab, University of West Florida, Pensacola, FL, United States; 2 Andrews Research & Education Foundation, Gulf Breeze, FL, United States; 3 Department of Mathematics, University of West Florida, Pensacola, FL, United States; 4 College of Medicine, Florida State University, Tallahassee, FL, United States

**Keywords:** functional performance, holistic training, military rehabilitation, pilot longitudinal study, tactical athlete

## Abstract

**Background:**

Tactical athletes face disproportionately high rates of musculoskeletal injury, with associated mental health consequences including fear-avoidance behavior and functional decline. While holistic programs integrating physical training and psychological support have shown promise for whole-person recovery, evidence for the durability of such benefits beyond the immediate post-intervention period in tactical populations remains limited.

**Objective:**

To quantify the physiological, psychological, and quality-of-life effects of the Game Plan program at baseline, immediately post-program, and at 90 days and 6 months follow-up.

**Methods:**

Twenty adult participants engaged in a four-week Game Plan program. Assessments included Body Composition Analysis, Mental Health Evaluation, Quality of Life Survey (QOLS), Tampa Scale of Kinesiophobia (TSK), EXOS Pillar Pre-Requisite Assessment, and a daily sleep log. Data was collected at baseline, post-program, and during remote follow-ups at 90 days and 6 months. This single-arm, observational pilot study was not designed to establish causation. Longitudinal changes were evaluated using linear mixed-effects models.

**Results:**

Twenty tactical athlete participants were enrolled in the study. All were white males with an average age of 41.4 (SD = 7.13) years old. A significant improvement in overall functioning was observed at 4 weeks compared with baseline (mean difference, 9.05; 95% CI: 2.20 to 15.90; *p* = 0.032; Cohen's d = 0.84). Kinesiophobia demonstrated a significant reduction at 90 days compared with baseline (mean difference, −4.40; 95% CI: −6.62 to −2.17; *p* < 0.001; Cohen's d = −1.37). Sleep duration showed a small but non-significant trend toward improvement at Week 4 compared with Week 1 (mean difference, 0.23; 95% CI: −0.03 to 0.49; Cohen's d = 0.22; *p* = 0.237. Larger studies are needed to confirm this observation. The Quality-of-Life domains, including Physical, Psychological, Social Health, and Environmental, showed no significant difference from baseline to 6 months.

**Conclusion:**

The four-week holistic training program with tactical athletes was associated with improvement in kinesiophobia, and overall functioning, with kinesiophobia gains persisting at six-month follow-up. A trend toward improved sleep duration was observed but did not reach statistical significance. The quality-of-life domains did not reach statistical significance. These pilot findings support further investigation through controlled trials to establish causal relationships.

## Introduction

Regular physical activity is widely recognized as a cornerstone of preventive health, with established benefits for cardiovascular function, muscular strength, endurance, flexibility, and balance (1). Beyond its physical effects, exercise exerts a significant influence on mental health, reducing symptoms of anxiety, depression, and stress while enhancing mood, cognitive function, and psychological resilience (2), defined here as the ability to adapt and maintain functional performance under chronic occupational stress. These benefits are amplified when exercise is delivered within a holistic framework that integrates physical, psychological, and lifestyle components. The application of a holistic framework to wellness is needed to support the needs of tactical athletes. Tactical athletes are those employed in the military, law enforcement, or first responder careers that require high levels of mental and physical exertion (3). These professions require a high physical demand threshold and mental health resilience as the professional often leads to prolific injury rates, which are detrimental to mental health (4). This is evidenced by 70% of military members developing a mental health disorder after sustaining an injury (5). Therefore, a holistic approach to the wellness of tactical athletics is needed to support the physical and mental well-being of these professionals.

The Game Plan training program was designed to operationalize this holistic model of well-being. Developed in collaboration with EXOS, a global leader in human performance coaching, the program combines evidence-based physical training with targeted mental health assessment, sleep monitoring, and personalized movement analysis. This approach aims to improve not only physical capacity but also psychosocial well-being and daily functioning of the tactical athlete population.

While the benefits of exercise are well-documented in both athletic and general populations, there is limited evidence of the durability of such benefits following short-term, intensive, multidisciplinary training. The Game Plan study addresses this gap by evaluating participants at baseline, immediately after a four-week intervention, and at two post-program intervals (90 days and 6 months) to assess sustained outcomes.

Given the exploratory and observational nature of this single-arm pilot design, findings are intended to be hypothesis-generating rather than confirmatory, and causal inference is not supported. The objective of this study was to evaluate if tactical athlete participants would demonstrate measurable improvements in physical health markers, psychological well-being, and self-perceived functional status that would persist for at least six months following program completion.

## Methods

Tactical athletes presenting to Andrews Research & Education Foundation to participate in the Game Plan holistic wellness program were recruited to participate in this prospective research project. This research project was reviewed and approved by Baptist Healthcare Institutional Review Board (IRB protocol # 2096251-5). The Game Plan program consisted of a four-week, residential onsite training experience administered by EXOS practitioners at the Andrews Research & Education Foundation facility. The program was structured around four pillars of holistic health: (1) mindset, incorporating targeted mental health evaluation and psychological skills training delivered by professionals; (2) nutrition, including individualized dietary assessment and education; (3) movement, comprising daily evidence-based physical training sessions encompassing mobility, strength, and conditioning components tailored to each participant's Pillar Pre-Requisite Assessment; and (4) recovery, which included sleep hygiene education and daily sleep monitoring. Sessions were conducted five days per week over the four-week period. Enrollment in the research study was voluntary and independent of enrollment in the Game Plan program. All eligible participants who enrolled in Game Plan during the study period consented to research participation, and no eligible participants declined. While several co-authors are affiliated with Andrews Research & Education Foundation, no co-authors served as Game Plan program participants or trainers.

Quality of life was assessed using the WHOQOL-BREF, a 26-item validated self-report instrument that evaluates four domains of quality of life, namely Physical Health, Psychological, Social Relationships, and Environmental Health, each scored on a transformed 0–100 scale, where higher scores indicate better quality of life (6). Kinesiophobia was assessed using the Tampa Scale of Kinesiophobia (TSK-17), a validated 17-item self-report measure of fear of movement and re-injury, scored from 17 to 68, with higher scores indicating greater kinesiophobia (7). Ninety days after completing the onsite Game Plan program, participants underwent the same Mental Health Assessment, and Kinesiophobia Assessment to evaluate whether the changes that occurred during the program were sustained after leaving the training facility. Overall functioning was assessed using the Single Assessment Numeric Evaluation (SANE). It is a validated single-item patient-reported outcome measure in which participants rate their current level of functioning on a scale of 0–100 relative to their baseline, with higher scores indicating better functioning (8). Participants also self-reported body measurements, including height and weight, and the mental health assessments were completed again remotely. These same evaluations were completed again six months after completing the Game Plan program to evaluate the sustainability of the improvements observed while on site for Game Plan training. Of the 20 participants enrolled at baseline, 20 completed the 4-week onsite program (100%), 16 completed the 90-day follow-up (80%), and 12 completed the 6-month follow-up (60%). Participant attrition was primarily attributable to active military deployment, which precluded completion of remote assessments. The drop out was non-random, concentrated among participants returning to active duty, and thus represents a potential source of attrition bias. This is acknowledged as a study limitation.

All analyses were performed using R (R Foundation for Statistical Computing). R packages used included lme4, lmerTest, emmeans, effectsize, tidyverse, gtsummary, and flextable. Continuous variables are reported as mean ± standard deviation (SD) with range, and categorical variables as counts with percentages. To evaluate longitudinal changes, linear mixed-effects models were used to account for repeated measures within participants. Linear mixed-effects models were specified *a priori* using time as the primary fixed effect, with age and body mass index (BMI) included as covariates. Time was modeled as a categorical variable, with baseline as the reference level for mental health outcomes and Week 1 as the reference for sleep outcomes. All models included a random intercept for subject to account for within-subject correlation. The primary outcome was overall functioning. Secondary outcomes included kinesiophobia, sleep quality, and sleep duration. Adjusted mean differences between timepoints were estimated using estimated marginal means, with pairwise comparisons performed relative to the reference timepoint. Holm correction was applied to account for multiple comparisons. Effect sizes were calculated as Cohen's d using model-derived variance estimates and are reported with 95% confidence intervals. Linear mixed-effects models were estimated using restricted maximum likelihood, allowing inclusion of participants with incomplete follow-up under the assumption that data were missing at random. Model assumptions, including normality and homoscedasticity of the residuals, were assessed and found acceptable. Confidence intervals are Wald-based, and all tests were two-sided with significance set at *p* < 0.05.

## Results

### Participant data

Twenty tactical athlete participants were enrolled in the study. All were white males (100%), with an average age of 41.4 (SD = 7.13) years old. The ages of participants ranged from 31 to 54 years. The average height (in) was 71.10 (SD = 3.08, range: 65–77 inches). The average weight (in pounds) was 206.01 (SD = 34.90, range: 165–305 lbs.) for the participants. Table 1 summarizes the participants' attributes.

**Table 1:** Participant characteristics.

Characteristic	*N* = 20
Age	41.40 ± 7.13 (31.00,54.00)
Gender	
Male	20 (100%)
Race	
White	20 (100%)
Ethnicity	
Hispanic	3 (15%)
Irish	1 (5.0%)
White	16 (80%)
Height (in)	71.10 ± 3.08 (65.00,77.00)
Weight (lbs)	206.01 ± 34.90 (165.00,305.00)

Data presented as Mean ± SD (Min,Max); *n* (%).

### Sleep results

**Figure 1:**
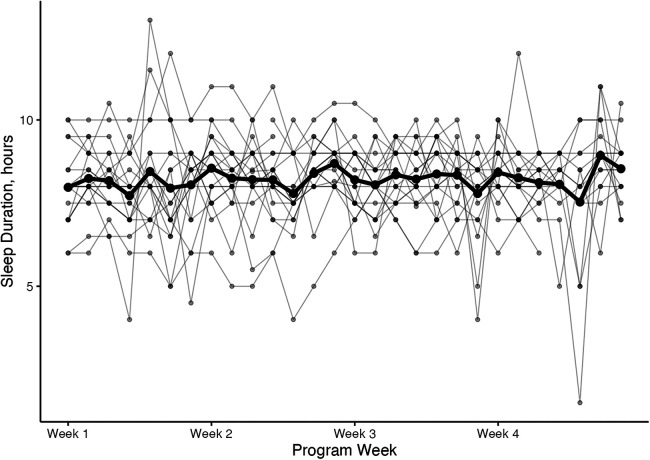
Weekly hours of sleep for participants.

**Figure 2:**
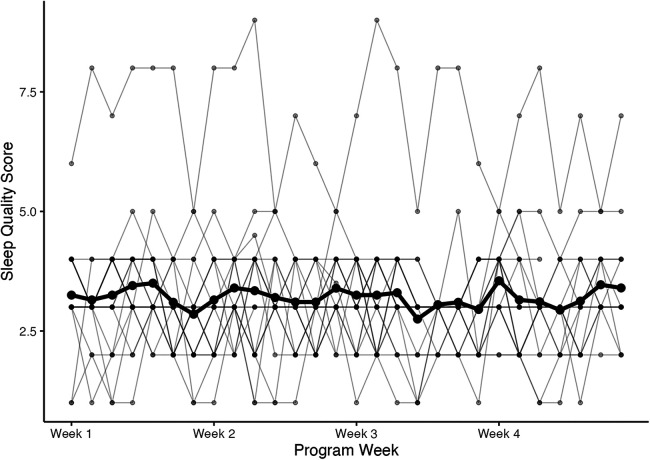
Sleep quality and disruptions for participants.

Sleep data was quantified using the daily quality, number of hours of sleep, and the number of times participants woke up after falling asleep for each participant (Table 2). Weekly averages were calculated, and Figures 1, 2 present the line graphs of the weekly averages of the number of sleep hours and quality across the 4 weeks of the program.

**Table 2:** Sleep outcomes by week.

Characteristic	Week 1	Week 2	Week 3	Week 4
Sleep Duration	8.1 ± 1.3; [4.0–13.0]	8.3 ± 1.2; [4.0–11.0]	8.2 ± 1.1; [4.0–10.5]	8.3 ± 1.3; [1.5–12.0]
Sleep Quality	3.2 ± 1.3; [1.0–8.0]	3.2 ± 1.3; [1.0–9.0]	3.1 ± 1.3; [1.0–9.0]	3.3 ± 1.2; [1.0–8.0]

Data present as Mean ± SD; [Min–Max].

A statistically significant change was not demonstrated in sleep duration across the four weeks of the program (all *p* ≥ 0.237). However, a small positive trend was observed at Week 4 compared with Week 1 (mean difference, 0.23; 95% CI: −0.03 to 0.49; Cohen's d = 0.22; *p* = 0.237), suggesting a possible trend toward improvement that warrants further investigation in adequately powered studies (Table 3). Sleep quality remained stable throughout the intervention period, with negligible effect sizes across all weeks (all *p* ≥ 0.579) (Table 4). The additional sleep metrics evidenced that participants were able to establish steady and consistent sleep patterns while completing the Game Plan Program.

**Table 3:** Adjusted Changes in Sleep Duration Compared with Week 1.

Comparison	Mean Difference (95% CI)	Effect Size, Cohen's d (95% CI)	*P*-Value
Week 2 - Week 1	0.22 (−0.03 to 0.47)	0.21 (−0.04 to 0.47)	.237
Week 3 - Week 1	0.11 (−0.14 to 0.35)	0.11 (−0.15 to 0.36)	.380
Week 4 - Week 1	0.23 (−0.03 to 0.49)	0.22 (−0.05 to 0.49)	.237

Values are presented as mean differences with 95% confidence intervals.

*P*-values adjusted using Holm correction for multiple comparisons.

Results from linear mixed-effects models comparing each week with Week 1. Values are presented as adjusted mean differences with 95% CI. Effect sizes are reported as Cohen's *d* with 95% CI. *P*-values were adjusted using the Holm method.

**Table 4:** Adjusted Changes in Sleep Quality Compared with Week 1.

Comparison	Mean Difference (95% CI)	Effect Size, Cohen's d (95% CI)	*P*-Value
Week 2 - Week 1	0.02 (−0.17 to 0.22)	0.03 (−0.23 to 0.28)	1.000
Week 3 - Week 1	−0.13 (−0.32 to 0.07)	−0.16 (−0.41 to 0.10)	.579
Week 4 - Week 1	0.01 (−0.19 to 0.21)	0.01 (−0.25 to 0.28)	1.000

Values are presented as mean differences with 95% confidence intervals.

*P*-values adjusted using Holm correction for multiple comparisons.

Results from linear mixed-effects models comparing each week with Week 1. Values are presented as adjusted mean differences with 95% CI. Effect sizes are reported as Cohen's *d* with 95% CI. *P*-values were adjusted using the Holm method.

Body metrics were analyzed at baseline and completion of the four-week Game Plan program. There was no statistical evidence to support differences in the BMI from Baseline to week 4. This suggests that participants’ BMI values remain relatively stable after 4 weeks in the program. The Wilcoxon signed rank test with continuity correction (*p* = 0.227; 95% CI [−0.1, 0.39]; (pseudo)median difference=0.15).

### Kinesiophobia

**Figure 3:**
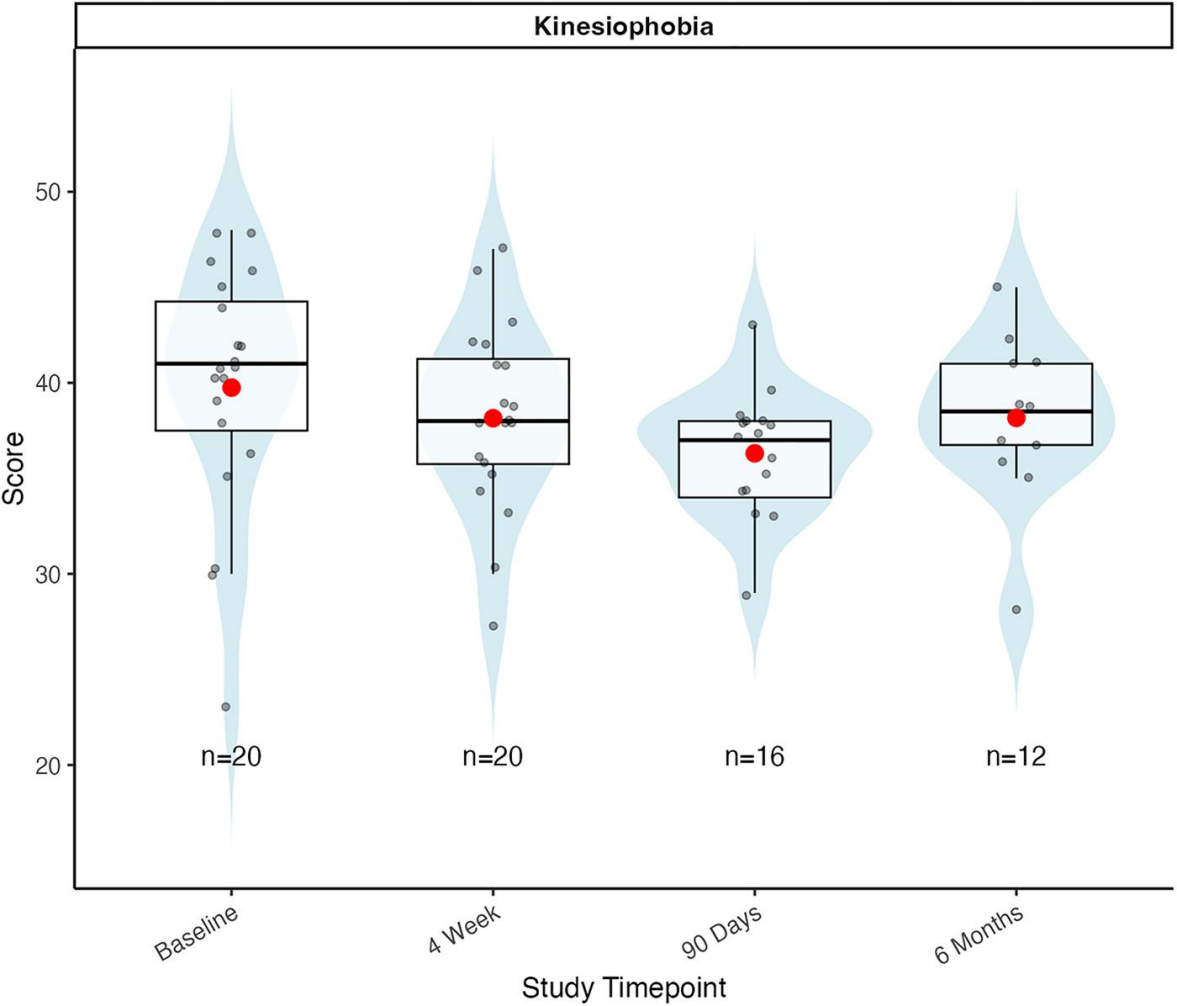
Participant results for kinesiophobia across evaluation time points.

Kinesiophobia was evaluated at baseline, 4 weeks, 90 days, and 6 months. A significant reduction in kinesiophobia was observed at 90 days compared with baseline (mean difference, −4.40; 95% CI: −6.62 to −2.17; *p* < 0.001; Cohen's d = −1.37), representing a large effect size (Table 5). A moderate reduction was also observed at 6 months (mean difference, −2.64; 95% CI: −5.11 to −0.18; Cohen's d = −0.82), though this did not reach statistical significance after Holm correction (*p* = 0.072). No significant change was observed at 4 weeks. Figure 3 presents the kinesiophobia scores for participants.

**Table 5:** Adjusted changes in kinesiophobia compared with baseline.

Comparison	Mean Difference (95% CI)	Effect Size, Cohen's d (95% CI)	*P*-Value
4 Week - Baseline	−1.60 (−3.65 to 0.45)	−0.50 (−1.17 to 0.17)	.122
90 Days - Baseline	−4.40 (−6.62 to −2.17)	−1.37 (−2.14 to −0.60)	<.001
6 Months - Baseline	−2.64 (−5.11 to −0.18)	−0.82 (−1.64 to −0.00)	.072

Values are presented as mean differences with 95% confidence intervals.

*P*-values adjusted using Holm correction for multiple comparisons.

Results from linear mixed-effects models comparing each follow-up timepoint with baseline. Values are presented as adjusted mean differences with 95% CI. Effect sizes are reported as Cohen's d with 95% CI. *P*-values were adjusted using the Holm method.

### Quality of life results

**Figure 4:**
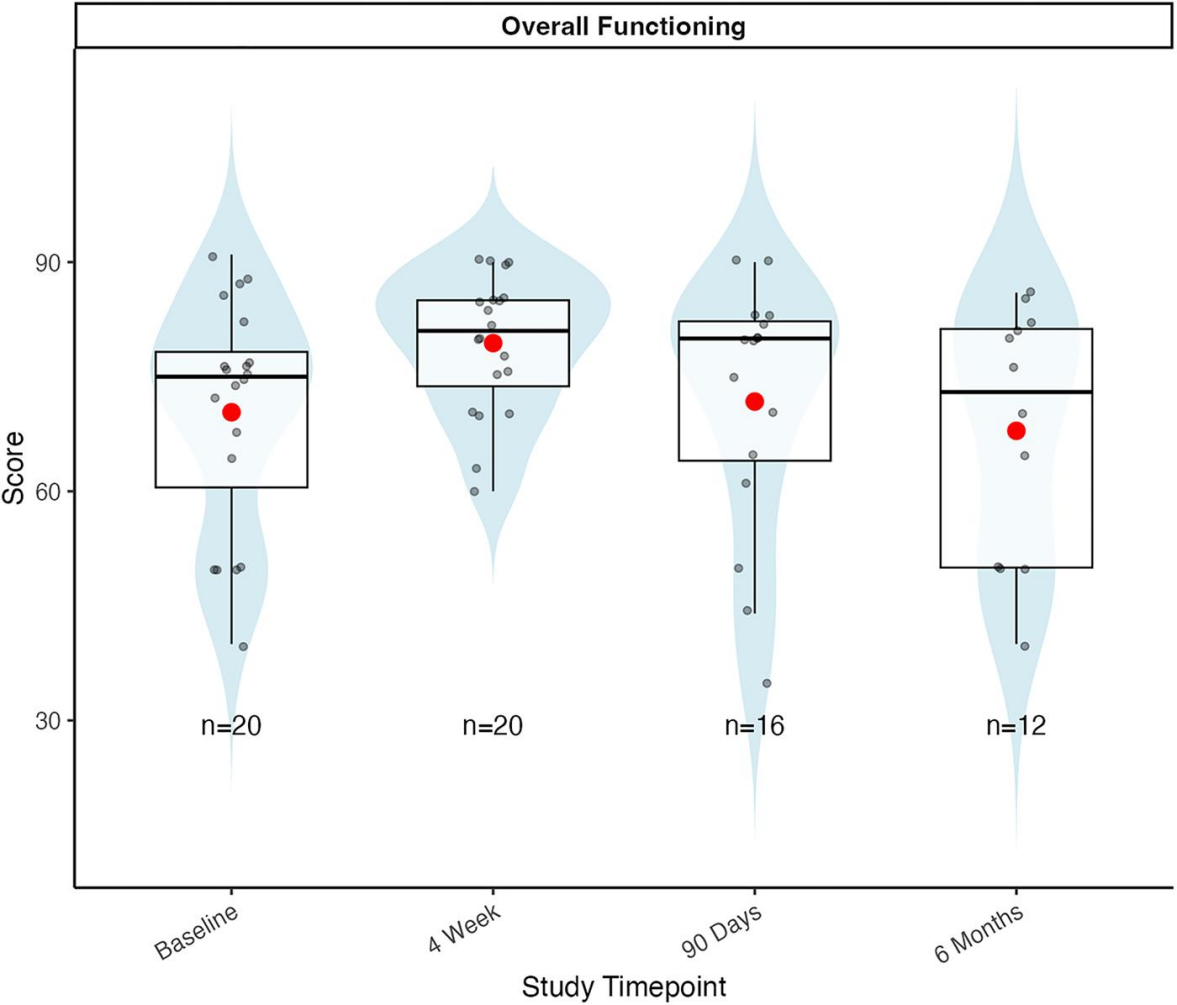
Overall functioning results across baseline, 4 weeks, 90 days, and 6 months evaluation time points.

The Quality-of-Life evaluation included domains to evaluate Physical, Psychological, Social Health, and Environmental Health. The data showed no significant difference from baseline to 6 months across the domains, as reported in Figure 4. The Wilcoxon signed rank test with continuity correction was employed. All *p*-values greater than 0.51 indicated no statistically significant differences. Table 6 reports the quality of life results.

**Table 6:** Descriptive statistics of quality of life, including mental health, for participants at baseline, at the 4-week mark (end of program), and at follow-up at 6 months.

	Time			
Characteristic	Baseline *N* = 20	4 Week *N* = 20	90 Days *N* = 16	6 Months *N* = 12
QOLS Physical	63.25 ± 13.83 (31.00,84.00)	-	-	64.17 ± 15.84 (31.00,81.00)
QOLS Psychological	69.62 ± 8.84 (48.80,81.00)	-	-	71.67 ± 13.47 (44.00,94.00)
Social Health	68.60 ± 15.30 (25.00,100.00)	-	-	70.83 ± 13.78 (50.00,100.00)
Environmental	76.53 ± 8.17 (60.00,88.00)	-	-	77.00 ± 12.68 (56.00,100.00)
Kinesiophobia	39.75 ± 6.43 (23.00,48.00)	38.15 ± 4.94 (27.00,47.00)	36.31 ± 3.28 (29.00,43.00)	38.17 ± 4.26 (28.00,45.00)
Overall Functioning	70.35 ± 14.90 (40.00,91.00)	79.40 ± 9.02 (60.00,90.00)	71.75 ± 16.46 (35.00,90.00)	67.92 ± 16.39 (40.00,86.00)

Data presented as Mean ± SD (Min,Max).

### Overall functioning results

**Figure 5:**
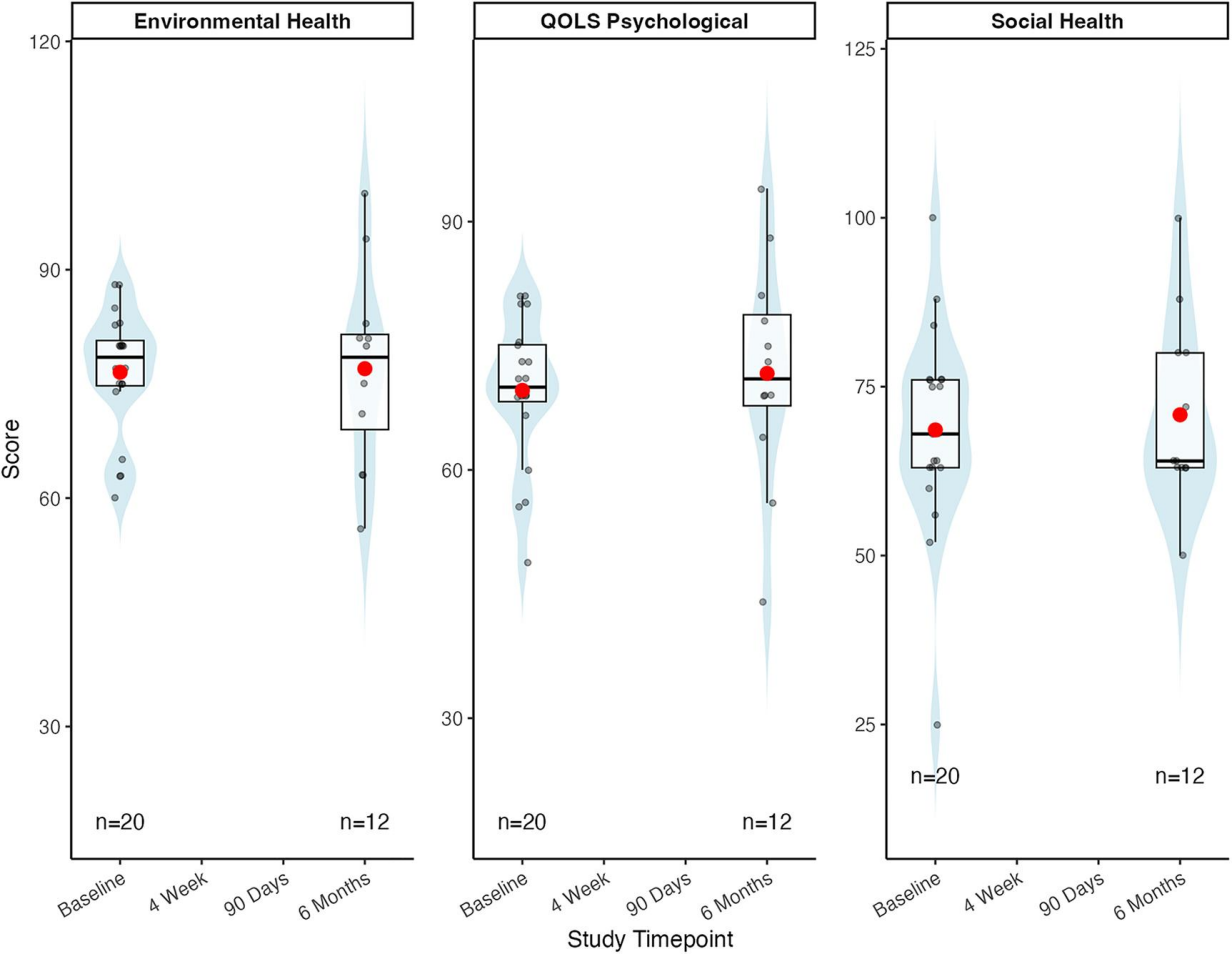
Quality of life scores for participants across the environment, physical, psychological, and social health domains.

A statistically significant improvement was observed in overall functioning at 4 weeks compared with baseline (mean difference, 9.05; 95% CI: 2.20 to 15.90; *p* = 0.032; Cohen's d = 0.84), representing a moderate-to-large effect size (Table 7). No statistically significant differences from baseline were observed at 90 days (mean difference, 2.23; 95% CI: −5.19 to 9.64; *p* = 1.000; Cohen's d = 0.21) or at 6 months (mean difference, −1.49; 95% CI: −9.71 to 6.72; *p* = 1.000; Cohen's d = −0.14), with small-to-negligible effect sizes at follow-up. Figure 5 reports the overall functioning results.

**Table 7:** Adjusted changes in overall functioning compared with baseline.

Comparison	Mean Difference (95% CI)	Effect Size, Cohen's d (95% CI)	*P*-Value
4 Week - Baseline	9.05 (2.20 to 15.90)	0.84 (0.16 to 1.52)	.032
90 Days - Baseline	2.23 (−5.19 to 9.64)	0.21 (−0.51 to 0.92)	1.000
6 Months - Baseline	−1.49 (−9.71 to 6.72)	−0.14 (−0.93 to 0.66)	1.000

Values are presented as mean differences with 95% confidence intervals.

*P*-values adjusted using Holm correction for multiple comparisons.

Results from linear mixed-effects models comparing each follow-up timepoint with baseline. Values are presented as adjusted mean differences with 95% confidence intervals (CI). Effect sizes are reported as Cohen's d with 95% CI. *P*-values were adjusted using the Holm method for multiple comparisons.

## Discussion

Tactical athletes are presented with physically taxing and emotionally burdened work conditions. The U.S. Armed Services active tactical athletes account for more than 866,00 annual clinic visits due to musculoskeletal injuries (9). Musculoskeletal injuries sustained by the tactical athlete population are one of the leading causes of dropout from military service (10–12). These alarming data support the need for change in the training and support of our military service providers. The Game Plan program provides a holistic approach to health for the tactical athlete that incorporates sleep, mental health, quality of life, and physical metrics into a comprehensive evaluation, which is used to create individualized training programs.

The holistic intervention included mental health support along with physical activity training. Regular physical activity has been linked to a wide range of health benefits, including improved cardiovascular function (13–15), increased muscular strength and endurance[2009 (16),], enhanced flexibility and balance (17), and reduced risk of chronic conditions such as obesity, diabetes, and cardiovascular diseases (18–22). These findings have provided the foundation for the evidence-based approach that the Game Plan training program utilizes to design its comprehensive exercise program. In addition to the well-established physical health benefits, exercise has also been extensively studied for its impact on emotional and mental well-being. Research suggests that engaging in regular exercise can significantly reduce symptoms of anxiety, depression, and stress, while improving overall mood and cognitive function (23, 24). This is attributed, in part, to the release of endorphins during physical activity, which act as natural mood elevators and contribute to a sense of well-being (25). However, the psychological benefits of exercise extend beyond neurobiological mechanisms and include improvements in self-efficacy and emotion regulation (26). These factors are particularly important in tactical athletes where physical capability and occupational performance are closely connected. Furthermore, recent evidence demonstrates that structured integrated physical interventions can simultaneously enhance emotion regulation and psychomotor skills (27), suggesting that the interaction between physical training and psychological development within holistic frameworks may be a key driver of the sustained psychological gains observed in the present study.

Notably, Quality-of-Life domains including Physical, Psychological, Social Health, and Environmental Health did not demonstrate statistically significant changes across the study period (all *p* > 0.51). Several explanations may account for these findings. Baseline QoL scores were in the moderate-to-high range, suggesting a potential ceiling effect that limited detectable improvement. This is a well-documented phenomenon in QoL measurement whereby instruments lose sensitivity when respondents cluster near the upper bound of the scale (28). The four-week program duration may be insufficient to produce measurable QoL change in a population accustomed to chronic high-demand occupational stress, as QoL in high-stress occupational groups has been shown to demonstrate resistance to short-term change (29). The reduced sample at six-month follow-up (*n* = 12) substantially limits statistical power for these comparisons, consistent with evidence that QoL measurements typically require considerably larger samples to detect meaningful change (30, 31). However, it is important to note that these findings should be interpreted alongside the positive outcomes rather than in isolation, and future studies with larger samples and longer follow-up periods are needed to clarify QoL trajectories in this population. It is important to distinguish the pattern of findings across physical and psychological outcome domains. Physical markers including BMI and sleep duration did not demonstrate statistically significant changes over the course of the program, suggesting that either the four weeks of structured training may be insufficient to produce detectable physiological adaptation in this population, or that a larger sample is required to detect such changes. In contrast, psychological outcomes, particularly kinesiophobia and overall functioning as measured by the SANE demonstrated meaningful improvements that were statistically significant at key timepoints and, in the case of kinesiophobia, persisted well beyond the intervention period. This dissociation suggests that the holistic components of the Game Plan program addressing fear-avoidance behavior, psychological skills, and mental health may confer more rapid and durable benefits than physical conditioning metrics alone. This finding may have direct implications for program design priorities in tactical athlete populations.

It is also worth noting that volunteer bias may influence sample characteristics in the larger study. Evidence suggests that individuals with fewer mental health challenges are more likely to participate in exercise programs (32). While in the current pilot study all eligible participants volunteered to participate and this could be partially due to their relatively high baseline scores, in a larger study the volunteer bias would raise the possibility that those who might benefit the most from a holistic intervention such as Game Plan would be least likely to volunteer.

Although the Game Plan program lasted only four weeks for the participants in this study, their holistic evaluation of health improved during training, and these improvements were sustained for six months after the training. The sustainability of changes serves as evidence that the self-care skills taught during the Game Plan onsite programming generalized to the home or combat settings, depending on where each participant transitioned to after completing the Game Plan program.

### Limitations

Several limitations that must be considered when interpreting the results of the study. The single-arm pre-post design without a control group for comparison poses limitations and causation cannot be inferred. Therefore, the observed changes may be associated with the Game Plan intervention but cannot be confidently attributed to it as the alternative explanations such as regression to mean or reduced occupational stressors cannot be ruled out. In addition, without *a priori* power analysis and sample size of 20 participants, this pilot study is likely underpowered. The findings, therefore, should be considered hypothesis generating and preliminary rather than conclusive. Moreover, the exclusive white male sample limits the generalizability to broader tactical, female, or racially diverse populations. Another limitation was the self-reporting of the follow up body measurements. This limits the objectivity of those metrics. Finally, 40% attrition at 6 months introduces potential survivorship bias.

## Conclusions

This pilot study observed associations between participation in a four-week holistic training program and improvements in kinesiophobia and overall functioning in a small sample of tactical athletes. Overall functioning demonstrated significant improvement at 4 weeks, while kinesiophobia reached significant reduction at 90 days and remained meaningfully lower at six-month follow-up. A trend toward improved sleep duration was observed during the program but did not reach statistical significance, and larger studies are needed to confirm this observation. Quality-of-life domains did not demonstrate significant change. These preliminary findings are hypothesis-generating and randomized controlled trials with larger, more diverse samples are needed to establish efficacy.

## Data Availability

The raw data supporting the conclusions of this article will be made available by the authors, without undue reservation.
